# Cataract

**Published:** 1890-12-27

**Authors:** 


### SURGERY.

### CATARACT.

In the days, now far away, when old people were young, Cooper, Tyrrell, Middlemore, and others were the authorities on diseases the eye, and especially on cataract. Now we have Swanzy, Macnamara, Berry, Carter, Frost, and others, many of whom have written books on the subject. The term cataract appears to have been originated in order to express the old idea that some kind of veil had fallen behind the pupil. Many years ago, Middlemore defined cataract to be "an imperfection or loss of vision dependent on an opacity of either the crystalline lens or its capsule, or of both." We are not aware that this definition has been improved upon by more recent writers. Of course the imperfection of vision may assume various forms. But, as a rule, there is gradually increasing dimness of vision, objects appear in a mist, and outlines are indistinct or distorted. In some cases there may be floating spots before the eyes. In others the person sees best when looking sideways. The progress of the malady may be quite painless, or there may be occasional pain, or headache, or irritation of the eye. These differences depend considerably on the variety of cataract, and on the progress or stage of the disease. As there are no blood vessels entering the lens, it was early believed that cataract is not an inflammatory condition. The lens was supposed to derive its nourishment by a process of imbibition, and that some deleterious agent was thus imbibed. Neither do we seem to have advanced very much on this ancient theory. For Macnamara attributes cataract to alterations in the constituents of the blood or to defective innervation. Carter and Frost remark that cataract is purely a degenerative change. The difference of symptoms may, however, be explained to a considerable extent by the nature and progress of the changes of structure which go on. For example, opacity may commence in the centre, or in the circumference of the lens, or it may proceed laterally, in the spaces and clefts between the lenticular fibres. These conditions would, of course, interfere with vision in different manners. A cataract may become more or less hard and irregular, which excites irritation and pain. Then there is the so-called black cataract, which depends upon infiltration of hematine into the opaque lens, the discoloration further adding to the defect of vision. Before the invention of the ophthalmoscope the most famous test for cataract was that known as Sanson's. This was based on the laws of reflection of light from curved surfaces. It consists in observing whether, when a candle flame is held near the eye and a little to one side of its optic axis, reflections can be seen from the two surfaces of the lens, as well as from the cornea. When the test is applied to the normal eye three images can be seen—two erect, and following in the same direction any movement given to the candle flame; and one inverted, which moves in the opposite direction to the object. The nearest erect image is bright, and is caused by reflection from the surface of the cornea. The next image is also erect though somewhat blurred, and is caused by reflection from the anterior surface of the lens. The third inverted image is reflected from the posterior concave surface of the lens, but it is fainter and small and not quite easily seen without the aid of a convex lens.

We have recalled these prominent features of cataract to mind, as a prelude to a paper recently appearing in the *New York Medical Record*, by Dr. Richard Kalish, ophthalmic surgeon to Charity Hospital, N.Y., and headed "The Arrest and Partial Resorption of Immature Cataract, with Restoration of Reading Power." Dr. Kalish asks, since non-traumatic cataract depends upon an interference with or deficient nourishment of the lens, how can an additional supply of nutrient material be furnished to the intra-ocular structures? Dr. Kalish's method consists of conjoined instillation and manipulation. He uses a mixture of equal parts of glycerine and a one per cent. solution of boric acid in rose water. If this causes too much irritation, that is, if it lasts longer than from five to twenty seconds, he reduces the proportion of glycerine. Conjoined with this instillation he employs a form of manipulation, not massage, conducted as follows: The patient is seated, with the head supported. Two drops of the solution are placed in each eye, and the operator standing behind the patient places both hands over the closed eyes, so that the tip of each middle finger rests upon the eye-ball at its nasal side, the index and ring finger falling into place beside the middle finger. With slight pressure upon the eye-ball, the three fingers are drawn outwards over the eye to the temporal side. This procedure is repeated from twenty to thirty times a minute, the stroking being in one direction only, and continued for ten minutes, when a second instillation of two drops into both eyes should be made, and manipulation carried on as before for ten minutes. The treatment is to be continued daily for a week, and then the interval between the instillations is to be lengthened to fifteen minutes, making the treatment three quarters of an hour in duration. After the treatment the face is cleaned with hot water. Dr. Kalish observes that the plan of treatment seems simple, and its application no difficult matter. "But in the hands of an inexperienced operator it cannot but be fraught with danger to the integrity of the delicately organised visual apparatus." Ill-regulated or too deep pressure is likely to do injury. Dr. Kalish gives the details of six cases treated in this manner, by which reading power was restored, and also states he has treated ten other cases with similar success. Manipulation alone has, however, failed. He also tried unsuccessfully *cineraria maritima* juice, as advised by Dr. Mercer some time ago in the *British Pharmaceutical Journal*. Pulsatilla in various forms was also tried, but without result. Castor oil was found ineffectual. Dr. Kalish, therefore, always uses the mixture described above.

Now the cure of cataract without operation has long been a desideratum eagerly sought for. It was long since asserted that friction over the globe, combined with some kind of medication, would cure or ameliorate cataract. In Heister's Surgery, published a hundred years ago, ether and electricity were recommended, and it may be mentioned that Dr. Kalish in his paper referred to above mentions that the galvanism may exert some beneficial influence. In other old works, the application of various stimulating vapours is advised. As regards internal medicines, Boerhaave stated "mercurius sæpe cataractas solvit"; and Van Swieten said corrosive sublimate was often administered "cum eventu felicissimo." Valentin recommended emetics! It must not, however, be forgotten that a few instances of undoubted spontaneous cure of senile cataract by absorption are on record. In an old treatise on cataract, Majendie is credited with quoting Gondret as stating that eventually lenticular opacity will be cured by absorption, and the dangers incident to an operation avoided. Dr. Kalish thinks he has solved the problem, and certainly if the treatment he advises is as successful generally as it seems to have proved in the author's hands, we may look for great advances in the treatment of cataract without operation. It must, however, be recollected that most ophthalmic surgeons do not believe that any relief can be brought about without operation. Carter and Frost say, "its arrest by any kind of medical treatment seems hardly to fall within the bounds of possibility." Swanzy remarks that "no external local applications nor internal medicine are of any avail."

The connection of cataract with other diseases is also touched upon by Dr. Kalish. Years ago it was remarked that cataract often occurred in gouty or rheumatic individuals, and Middlemore expressly dwelt upon this. There has also been supposed to be a "diabetic cataract." But Macnamara remarks that it is a mistake to suppose persons with diabetes are particularly subject to cataract. It has also been supposed to be connected with albuminuria. But as Mr. Berry has observed, "no certain connection between senile cataract and any other disease has been definitely established."

In connection with the subject we may refer to a paper by Dr. Richmond Lennox, surgeon to the Brooklyn Eye and Ear Hospital, on the composition of the human lens. In the cataractous lenses, the total weight is less than in the clear, and diminishes with age. This appears to depend on the loss of water. As the results of his investigations, the author concludes that "a cataract is not likely to be more solid because the lens in which the change has occurred is an old one, and any treatment based on such supposition is fallacious and unsound." We must be guided in deciding as to operation on immature cataracts by the circumstances of the individual case.

